# The effects of topical sodium cromoglicate on itch and flare in human skin induced by intradermal histamine: a randomised double-blind vehicle controlled intra-subject design trial

**DOI:** 10.1186/1756-0500-4-47

**Published:** 2011-03-07

**Authors:** Alan M Edwards, Michael T Stevens, Martin K Church

**Affiliations:** 1 The David Hide Asthma and Allergy Research Centre, St Mary's Hospital, Newport. PO30 5TG. Isle of Wight. UK; 2 EMStat Ltd., 191, Markfield Lane, Newtown Linford, Leicestershire LE67 9PQ. UK; 3 Department of Dermatology and Allergy, Allergie-Centrum-Charité, Charité-Universitätsmedizin, Berlin, GERMANY and School of Infection, Inflammation and Repair, Faculty of Medicine, University of Southampton, Southampton General Hospital, Southampton SO16 6YD. UK

## Abstract

**Background:**

Itch is a prominent feature of many skin diseases, particularly atopic dermatitis and cutaneous mastocytosis. Sodium cromoglicate (SCG), a chromone developed for the treatment of allergic disease has been shown to reduce the severity of itch when applied topically to subjects with atopic dermatitis. The aim of this study was to investigate whether topical sodium cromoglicate can reduce the severity of itch induced by intradermal histamine.

**Methods:**

SCG was introduced into the skin of healthy volunteers both by iontophoresis and by topical application using a new 4% cutaneous emulsion (Altoderm™). The skin was then challenged with intradermal histamine. Measurements were made of severity of itch, size of wheal and flare and change in blood flux

**Results:**

SCG significantly reduced the severity of itch (*P *= 0.0045) and flare (*P *= 0.0143) when delivered by iontophoresis. SCG 4% cutaneous emulsion significantly reduced severity of itch (*P *= 0.024) and flare (*P *= 0.015) in atopic subjects. Trend analysis showed increasing effect on itch with increased concentrations of SCG, which was significant (*P *= 0.046). There were no effects on wheal or blood flux.

**Conclusions:**

Topically applied SCG, administered in a new cutaneous emulsion base, significantly reduced the itch and flare caused by intradermal histamine. The effect was greatest in atopic subjects and increased with the concentration of SCG in the emulsion.

**Trial registration:**

ISRCTN35671014

## Background

Itch is a prominent feature of many skin diseases, particularly atopic dermatitis and cutaneous mastocytosis. Itch can be triggered by local, systemic, peripheral or central stimuli. The transmission of itch to the brain is the function of a sub-population of the dense nerve network in the skin, unmyelinated C-neurons, which are histamine sensitive [1]. Intradermal injection of histamine into human skin results in a wheal, flare, erythema and itch. It therefore represents a useful model for investigating treatments that might reduce itch severity.

Sodium cromoglicate (SCG), a chromone developed in the 1960's as an inhaled powder for the treatment of asthma [2] has subsequently been used for the treatment of allergic rhinitis, allergic conjunctivitis, food allergy and systemic mastocytosis. A number of studies have investigated the effect of topically applied SCG in atopic dermatitis [3-7]. Although variable effects have been reported, probably because of the hydrophilic nature of SCG that limits its penetration in intact skin [6], several have found a significant reduction of itch [3-5].

While mast cell stabilization was proposed as the primary mode of action of SCG in the treatment of asthma [2], this is unlikely to be its primary mechanism of action in the skin for two reasons. First, SCG does not inhibit histamine release from human skin mast cells in experimental models *in vitro *[8] or *in vivo *[9]. Second, while it can reduce both histamine-induced itch and flare, it does not reduce the wheal [10]. The most likely mechanism of action is to prevent the activation of sensory nerves. This was initially proposed from airway studies in humans [11,12] and experimental animals [13]. This proposal is supported by a study with the related chromone, nedocromil sodium, which, when introduced into the skin by iontophoresis, causes inhibition of histamine-induced itch and flare but has no effect on the wheal [14]. Further studies with nedocromil sodium have shown it to reduce sensory nerve activation, critical in causing both itch and flare by inhibiting a Na^+^/K^+^/2Cl^- ^co-transporter in the sensory nerve membrane [15].

Our hypothesis was that SCG will have a similar effect on histamine-induced itch and flare as that demonstrated by nedocromil sodium, both when introduced into the skin by iontophoresis and after applying it in a new cutaneous emulsion (Altoderm™), designed to optimise the penetration of SCG into the skin. We further hypothesised that the response would be dose related and the effect would be greater in atopic subjects.

## Methods

Thirty-five healthy volunteers (15 non-atopic and 20 atopic), with no sign of any skin disease participated in this study, which was approved by the Southampton and South West Hampshire Research Ethics Committee and was conducted according to the Declaration of Helsinki. All gave written informed consent to participate in the studies.

At the first visit, the suitability of the subjects to take part in the study was assessed and whether they met the inclusion and exclusion criteria. Exclusion criteria included pregnancy, the presence of skin disease and taking drugs that might interfere with the study, including corticosteroids, antihistamines, antidepressants and psychotropic drugs. Atopic status was determined by the presence of a history of allergic disease, and a positive skin prick test to house dust mite, grass pollen or cat allergen. For the skin prick tests histamine and sterile saline were used as positive and negative controls. A positive response was defined as a wheal of more than 3 mm greater than the negative control.

### Iontophoresis

SCG (a gift from Hewlett Healthcare Ltd, Derby, UK.) was introduced into the skin using iontophoresis (MIC1-e, Moor Instruments Ltd, Axminster, Devon, UK). The chamber was fixed to the skin, filled with a 4% solution of SCG in reverse osmosis purified water and a total charge of 8 mC applied over a period of 40 seconds. It was calculated that this charge would introduce 38.8 μg of SCG into the skin over an area of approximately 0.8 cm^2 ^(1 cm diameter). At control sites, the iontophoresis chamber was filled with reverse osmosis purified water. Readings of blood flux were taken before and 5 minutes after iontophoresis to assess any drug- or iontophoresis-induced dermal vasodilatation.

### SCG cutaneous emulsion

Subjects were supplied with 150 ml bottles containing either the cutaneous emulsion base alone (vehicle) or the emulsion with SCG at 1%, 2% or 4% w/w concentration (supplied by Hewlett Healthcare Ltd). The emulsions were identical in appearance and perception after application to the skin.

### Histamine challenge

Histamine (Sigma, Poole, Dorset, UK) dissolved in sterile saline (0.9% NaCl) was injected intradermally (20 μl of 1 μM or 300 nM) into the centre of the iontophoresis sites or sites to which cutaneous emulsion had been applied, 10 minutes after iontophoresis or 10 minutes after the last application of the cutaneous emulsion. As batches of histamine vary in their biological potency, two concentrations of histamine were used so that the most appropriate could be selected.

### Scanning Laser Doppler Imaging

Wheal and flare areas and blood flux, indicative of dermal perfusion up to a depth of 1 mm, were assessed in the skin using scanning laser Doppler imaging (Moor LDI, Moor Instruments Ltd, Axminster, Devon, UK). Ten minutes after histamine injection, an area of skin of 5 cm square was scanned giving ~16,000 data points for analysis. Changes in weal and flare areas may be measured to an accuracy of ± 0.05 cm^2 ^and changes in perfusion to ± 5%.

### Planimetry

While scanning laser Doppler imaging is ideal for assessing areas of flare and blood flow, it is less accurate in determining the area of a wheal. Consequently, planimetry, in which wheal areas were calculated from traces of their outlines on acetate sheet, was used to confirm wheal size.

### Severity of itch

Immediately following histamine injection, subjects recorded the severity of itch on a 100 mm Visual Analogue Scale (VAS) every 20 seconds for 5 minutes.

### Preliminary investigation

On the first experimental day, subjects were asked to lie down for 10 minutes prior to the commencement of the study. The volar aspect of both forearms was wiped clean with antiseptic wipes (Sterets) and left to dry. Four sites, two on the volar aspect of each forearm, one for 1 μM histamine and the other for 300 nM histamine, were selected for the study. The VAS score for itch for 1 μM histamine, 33.97 ± 5.35 mm, was greater than that for 300 nM, 28.07 ± 4.25 mm. As a consequence, only the results from the 1 μM histamine sites were used for further calculations.

Three studies were conducted

### Study 1: Iontophoresis study

Baseline blood flux was measured by scanning laser Doppler imaging immediately before introduction of 4% sodium cromoglicate in aqueous solution or reversed osmosis purified water (ROW, control), were introduced into the skin using iontophoresis. The order and sites of iontophoresis of sodium cromoglicate and water were randomised with the restriction that the treatments were equally distributed between sites on the upper and lower forearm. Five minutes after iontophoresis, blood flux was reassessed using scanning laser Doppler imaging. Ten minutes after iontophoresis, 20 μl of 1 μM of histamine was injected intradermally and the subjects began to record the severity of itch on a VAS. Ten minutes after histamine injection, the area of the weal was measured using planimetry and the area and intensity of the flare measured using scanning laser Doppler imaging.

The techniques used for this study were similar to those described in the original study with nedocromil sodium [13].

### Study 2: Effect of 4% SCG cutaneous emulsion

Subjects were given 2 bottles of emulsion labelled L1 and R1. Emulsion L1 was used on the left forearm and emulsion R1 on the right. The containers contained either 4% sodium cromoglicate emulsion or the matching vehicle. Subjects were instructed to massage 1 ml of the emulsion into two areas of each forearm, each approximately 10 cm sq, delineated with a marker pen, four times a day for 3 days prior to the next experimental visit. On the experimental day, the last application of the emulsion was made 10 minutes before the injection of 20 μl of 1 μM of histamine. Recordings of itch, wheal and flare made as in study 1. All interventions were administered double-blind and randomised.

### Study 3: Comparison of 1%, 2% and 4% SCG cutaneous emulsions

Subjects were supplied with four bottles of emulsions labelled A, B C and D. Subjects were instructed to massage 1 ml of emulsion A into an area of approximately of 10 cm^2^, delineated with a marker pen, on the upper part of the volar surface of the left forearm four times a day for 3 days prior to the next experimental day. In a similar manner, emulsion B should be applied to the lower part of the left forearm, emulsion C to the upper part of the right forearm and emulsion D to the lower part of the right forearm. On the experimental day, the procedures followed were the same as in Study 2 with the last application of the emulsions being made 10 minutes before the injection of 20 μl of 1 μM of histamine. The containers contained 1%, 2% or 4% SCG emulsion or the matching vehicle. Treatments were randomised and administered double-blind.

### Statistical methods

All data are shown descriptively as means ± SEM for each treatment. For VAS scores for itch, for each subject the mean of all available values over the 5-minute period was used. A two-sided *P*-value of 0.05 was taken to indicate statistical significance in any statistical testing.

For study 1, a generalised linear model was fitted to the data incorporating atopic status, subject (within atopic status), treatment and treatment x atopic status interaction. If the interaction was not statistically significant then this was dropped from the model. The least square means for sodium cromoglicate and water are presented together with the difference and 95% confidence interval for the difference.

For study 2, the same method and model was used and the contrast SCG 4% emulsion and vehicle presented.

For study 3, a generalised linear model was fitted to the data incorporating subject and treatment. If the overall treatment effect was statistically significant then pair-wise comparisons of each dose versus vehicle were carried out. In addition, since the treatment groups consisted of increasing concentrations of SCG, concentration (0, 1, 2, 4) was included in the model as a covariate (with the subject factor only) in order to provide a test of linear trend.

Since both studies 2 and 3 contained within subject assessments of 4% concentration of SCG emulsion and vehicle, a combined analysis using only atopic subjects was carried out. A generalised linear model was fitted to the data incorporating study, subject (within study), treatment and treatment x study interaction. If the interaction was not statistically significant then this was dropped from the model. The least square means for SCG emulsion and vehicle are presented together with the difference and 95% confidence interval for the difference.

Checks that the underlying data were normally distributed were undertaken. The formal Shapiro-Wilk's test was satisfactory in all 8 analyses of both itch and flare. Similarly, the distribution of residuals and normal probability plots gave no concern and hence we are confident that the parametric models used for the analyses are appropriate.

## Results

### Study 1

Sixteen subjects, 8 atopic and 8 non-atopic, were included in study 1. The results for itch and flare are summarised descriptively and analysed statistically in Table 1.

**Table 1:** Effect of 4% SCG and reversed osmosis water (ROW) administered by iontophoresis on the itch and flare responses to intradermal histamine

Symptom (Measure)	SCG Mean ± SEM	ROW Mean ± SEM	SCG Least square mean	ROW Least square mean	Least square mean difference (95%CI)	P value
Itch (VAS mm)	23.24 ± 4.24	33.96 ± 5.35	23.24	33.96	10.72 (3.87,17.57	0.0045
Flare (cm^2^)	16.28 ± 3.53	21.68 ± 3.35	16.55	21.94	5.39 (1.29,2.50	0.0143

The interaction between treatment and atopic status was not statistically significant and was dropped from the model for both symptoms. Atopic status was not statistically significant for either symptom.

However, for the treatment effect, the results show statistically significant inhibitions by SCG of the itch response and flare area.

### Study 2

Twelve subjects, 5 atopic and 7 non-atopic, were included in study 2. The results for itch and flare are summarised descriptively and analysed statistically in Table 2.

**Table 2:** Effect of 4% SCG emulsion and vehicle on the itch and flare responses to intradermal histamine

Symptom (measure)	SCG 4% emulsion Mean ± SEM	Vehicle emulsion Mean ± SEM	SCG Least square mean	Vehicle square mean	Least square mean difference (95%CI)	P value
Itch (VAS mm)	17.39 ± 3.82	20.29 ± 5.09	18.56	21.46	2.90 (-1.68,7.48)	0.19
Flare (cm^2^)	22.96 ± 3.14	36.36 ± 3.94	23.12	36.52	13.40 (6.35,20.45)	0.0017

The interaction between treatment and atopic status was not statistically significant and was dropped from the model for both symptoms. Atopic status was not statistically significant for either symptom.

For the treatment effect in this study in which the treatments were applied as topical cutaneous emulsions, the results again show inhibitions by sodium cromoglicate of itch and flare area. However, only the effect for flare area is statistically significant

### Study 3

Seven subjects, all atopic, were included in study 3. The results for itch and flare are shown in Table 3.

**Table 3:** Comparison of 1%, 2% and 4% SCG emulsions and vehicle on the itch and flare responses to intradermal histamine

Symptom (n)	Vehicle emulsion Mean ± SEM	SCG 1% Mean ± SEM	SCG 2% Mean ± SEM	SCG 4% Mean ± SEM	P value
Itch (7)	20.89 ± 8.09	22.01 ± 8.46	18.7 ± 8.28	14.06 ± 4.08	P = 0.22
Flare (7)	19.37 ± 2.52	21.27 ± 3.05	26.87 ± 1.91	14.39 ± 1.45	P = 0.013

In this study 1% SCG cutaneous emulsion did not inhibit the itch response (-5% inhibition) while the 2% and 4%cutaneous emulsions inhibited the response by 10% and 33% respectively. Against the flare response, neither 1% nor 2% SCG inhibited the response (-10% and -39% inhibitions respectively) while the 4%cutaneous emulsion inhibited the response by 26%. There was a statistically significant difference between treatments for flare area but not for the itch response.

**Figure 1:**
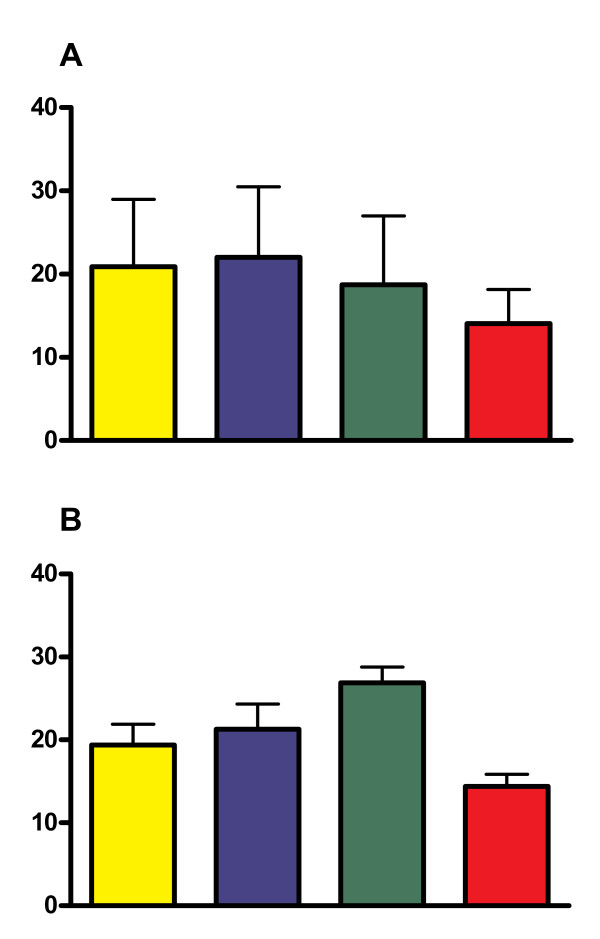
**Effect of SCG emulsions and vehicle on A. itch and B. flare**. The effect of different concentrations of SCG cutaneous emulsion and the vehicle emulsion on A. the severity of itch and B. the area of flare induced by intradermal histamine. Yellow columns = vehicle emulsion. Blue columns = SCG 1% concentration. Green columns = SCG 2% concentration. Red columns = SCG 4% concentration. Y axis: Severity of itch measured using 100 mm Visual Analogue Scale. Flare area measured in cm^2^. Statistical Analysis for linear trend, p = 0.046 for itch, p = 0.22 for flare area.

The test for trend with increasing concentration was statistically significant for itch response (*P *= 0.046), but not for flare area (*P *= 0.22), thus suggesting an increasing inhibition of itch response with increasing concentration of SCG (Figure 1).

### Combined analysis: SCG 4% v vehicle

SCG 4% cutaneous emulsion and vehicle were investigated in both studies 2 and 3. In order to provide a more precise comparison of the effects of SCG 4% compared to vehicle, the 5 atopic subjects from study 2 were analysed together the 7 subjects (all atopic) from study 3. Hence a total of 12 atopic subjects were included in this analysis. The results for itch and flare are summarised descriptively and analysed statistically in Table 4.

**Table 4:** Effect of 4% SCG emulsion and vehicle on the itch and flare responses to intradermal histamine in atopic subjects from studies 2 and 3

Symptom (measure)	SCG 4% emulsion Mean ± SEM	Vehicle emulsion Mean ± SEM	SCG Least square mean	Vehicle Least square mean	Least square mean difference (95%CI)	P value
Itch (VAS mm)	17.88 ± 4.32	25.00 ± 6.34	18.67	25.79	7.13 (1.12,13.13)	0.024
Flare (cm^2^)	19.11 ± 2.98	26.93 ± 4.02	20.33	28.16	7.82 (3.72,11.93)	0.0015

The interaction between treatment and study was not statistically significant and was dropped from the model for both symptoms.

**Figure 2:**
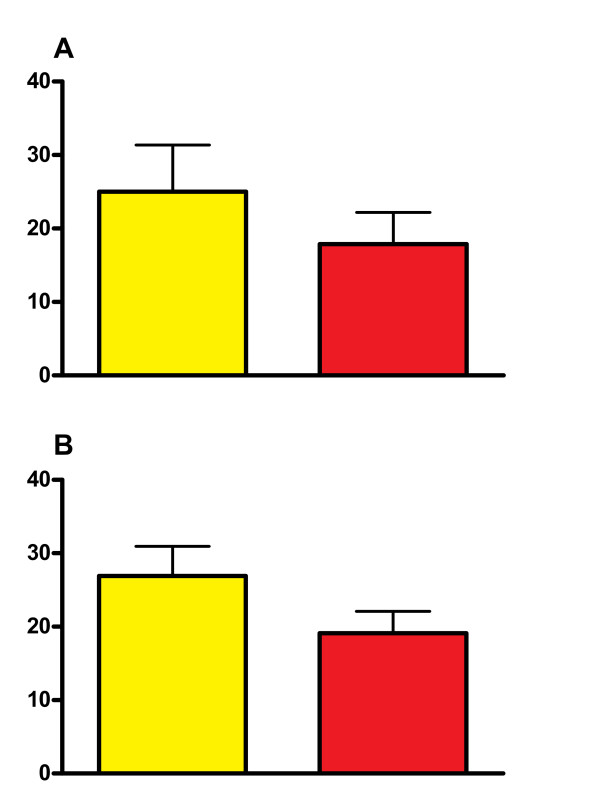
**Effect of SCG 4% cutaneous emulsion and vehicle on itch and flare in atopic subjects**. The effect of 4% SCG cutaneous emulsion and the vehicle emulsion on A. the severity of itch and B. the area of flare induced by intradermal histamine in atopic healthy subjects. Yellow columns = vehicle emulsion. Red columns = SCG 4% concentration. Y axis. Severity of itch measured using 100 mm Visual Analogue Scale. Flare area measured in cm^2^. Statistical Analysis for differences, p = 0.024 for itch, p = 0.0016 for flare area.

The treatment effect was statistically significant for itch response and flare area, with SCG 4% showing inhibition compared to vehicle in the combined analysis of the atopic subjects (Figure 2).

There was no effect on the size of the wheal or blood flux in any of the studies. These results are not shown.

## Discussion

The results in study 1 of these challenge experiments demonstrate the inhibition of the itch and flare responses by SCG but with no effect on the wheal or blood flux are in line with the previous observations with the related chromone, nedocromil sodium [14,15].

The numbers in each individual study were small and it was necessary to combine the results from the atopic subjects in study 2 and study 3 to show that this SCG emulsion is an effective anti-itch agent in this group of subjects. This also shows in this formulation the drug is able to penetrate intact skin in sufficient quantities to have clinically demonstrable effects.

In study 3, sodium cromoglicate at 1%, 2% and 4% concentrations gave inconsistent effects against the flare responses. However, there was statistical evidence to support an increasing effect on itch with increasing concentration of sodium cromoglicate. The greatest inhibition for both symptoms was seen with 4% concentration. The cutaneous emulsion used in this study, is a patented formulation for topical use containing 4% concentration of SCG in a novel emollient base designed to improve the absorption through the skin of the water soluble active ingredient SCG.

These results are supported by the results from a recent study, in which subjects were challenged with either intradermal allergen, intradermal codeine or intra-dermal histamine after application of a placebo aqueous cream, and creams containing 0.2%, 1%, and 4% SCG [9]. No intervention had any effect on wheal size or temperature change. The greatest effect on pruritus (itch) was in the allergic group challenged with allergen in whom all SCG treatments had a significant effect. In the non-allergic subjects challenged with histamine, only the 4% concentration was significantly better than placebo.

SCG has no anti-histaminic activity [2]. Our unit has shown that the drug is ineffective in preventing the release of histamine from dispersed skin mast cells derived from the foreskin of children [8]. It is unlikely that in this study SCG is acting as a mast-cell stabiliser. In the study in which the chromone NS was compared with frusemide and bumetanide [15], all significantly inhibited the itch and flare responses. The neurogenic responses, itch and flare, could possibly result from sensory nerve activation and axonal conduction, neuropeptide release, activation of neuropeptide receptors on blood vessels and the ability of the vasculature to respond to stimulation. As none of the drugs had any effect on blood flux, it is unlikely that the mechanism of action is on neuropeptide release, activation of neuropeptide receptors or a direct effect on the vasculature. We reasoned and concluded that all three drugs inhibited sensory nerve activation.

## Conclusions

We conclude that the 4% SCG cutaneous emulsion used in these studies will be useful in skin diseases such as atopic dermatitis (eczema) and cutaneous mastocytosis where itching is a predominant feature.

## Abbreviations

SCG: sodium cromoglicate; VAS: Visual Analogue Scale.

## Competing interests

AME and MTS are consultants to the manufacturers of Altoderm, Thornton & Ross Ltd. and were employees of the originators of sodium cromoglicate, Fisons Pharmaceuticals Ltd. MKC has received financial reward for lectures and consultancy from Glaxo Smith Kline, UK; UCB Pharma, Switzerland; Uriach Pharma, Spain; FAES Pharma, Spain; Almirall Pharma, Spain and Thornton & Ross Ltd. There is no conflict between these.

## Authors' contributions

AME conceived the study, contributed to the design, was partially responsible for the interpretation of the data and wrote the manuscript. MTS was responsible for the statistical analysis of the data, and has helped in the production of the manuscript. MKC had overall responsibility for the conception and design of the study, and the conduct of the study, and helped to draft the manuscript. All authors have read and approved the final manuscript.
